# Hemorrhagiectatic carcinoma: an uncommon clinical presentation of cutaneous metastasis from pulmonary adenocarcinoma^؆^

**DOI:** 10.1016/j.abd.2025.501286

**Published:** 2026-01-15

**Authors:** Nelson Lobos-Guede, Dan Hartmann, Felipe Carvajal Villarroel, Paloma Matus Concha, Catalina Silva-Hirschberg, Magdalena Delgado Barros

**Affiliations:** a Department of Head and Neck Surgery, Dermato-Oncology Service, National Cancer Institute, Santiago, Chile; b Department of Dermatology, Faculty of Medicine, Universidad de Chile, Santiago, Chile; c Department of Radiotherapy, National Cancer Institute, Santiago, Chile; d Department of Dermatology, Faculty of Medicine, Clínica Alemana, Universidad del Desarrollo, Santiago, Chile; e Department of Pathology, National Cancer Institute, Santiago, Chile

Dear Editor,

Cutaneous Metastases (CM) are rare and occur in 1%–5% of patients with internal malignancies. Although they can sometimes be the first manifestation of a primary cancer, they are typically detected in patients with a known history of malignancy. On average, the interval between the primary tumor diagnosis and the appearance of cutaneous metastases is 2.9 years.^1^ The most common primary tumors that give rise to CM are melanoma and adenocarcinoma of the breast and lung.^1^, ^2^, ^3^ In lung cancer, adenocarcinoma is the subtype with the highest frequency of cutaneous metastases (2.95%), surpassing squamous cell carcinoma (1.16%) and small cell carcinoma (0.81%).^1^ CM can present pleomorphic, with dermal nodules being the most common manifestation. A rare subtype is hemorrhagic carcinoma, characterized by violaceous, indurated plaques with tumor infiltration in blood vessels and lymphatics.^1^, ^3^, ^4^

We present a case of a 58-year-old woman with a diagnosis of primary pulmonary adenocarcinoma and brain metastases in stage IV. She received combined treatment with surgery, radiotherapy, and conventional chemotherapy. Due to lymph node progression during follow-up, Nivolumab was started, completing 8 cycles with poor response. She developed progressively extensive violaceous-purpuric plaques with some infiltrated areas on the left hemiface, neck, sternum, shoulders, breasts, and axillae, associated with pruritus and burning (Fig. 1A‒D). She was referred to the dermatology-oncology service with a suspected adverse reaction to Nivolumab, and an incisional biopsy showed dermis infiltrated by nests of atypical epithelial cells with extensive permeation in blood vessels and lymphatics (Fig. 2A‒B). Immunohistochemistry showed strong positivity for cytokeratin 7, TTF1, and NapsinA (Fig. 2C‒E), confirming the diagnosis of cutaneous metastasis from poorly differentiated lung adenocarcinoma. Brachytherapy (BT) was performed, leading to a significant reduction in lesion extent, infiltration, and symptoms. The patient underwent multiple courses of radiotherapy throughout the disease course. Initially, Whole-Brain Radiotherapy (WBRT) was administered at 30 Gy in 10 fractions, followed by a boost to the surgical bed of 36 Gy in 10 fractions after resection of brain metastases. For painful cervical lymphadenopathy, palliative External Beam Radiotherapy (EBRT) was delivered at 20 Gy in 4 fractions. Cutaneous metastases involving the hemiface, neck, thorax, shoulders, and axillae were treated with surface brachytherapy using a custom-made mold, delivering 20 Gy in 4 fractions, resulting in significant clinical improvement. Additionally, symptomatic thoracic bone metastases received a single-fraction palliative dose of 8 Gy via teletherapy. No electron-based teletherapy was used for skin lesions. Subsequently, Erlotinib was prescribed, and the cutaneous lesions continued to improve (Fig. 3A‒C). The disease remained stable temporarily for a few months before progressing with bone metastasis and progressive deterioration, leading to the patient’s death.

**Figure 1:**
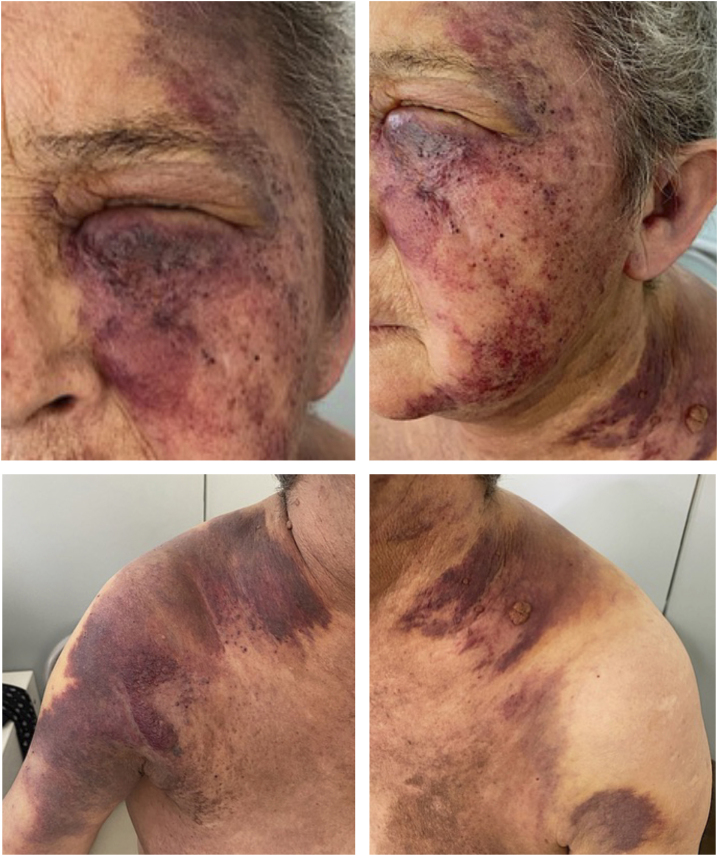
presence of an extensive violaceous-purpuric plaques with some infiltrated areas on the left hemiface, neck, sternum, shoulders, breasts and axillae.

**Figure 2:**
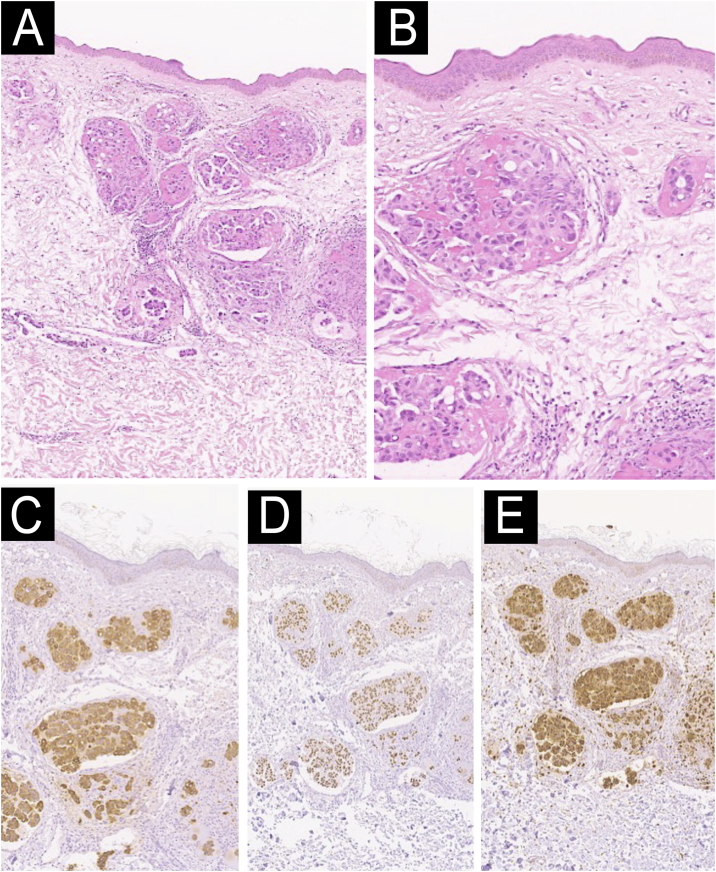
(A) Hematoxylin and eosin staining reveals extensive malignant infiltration of the dermis and subcutaneous tissue by atypical epithelial cells arranged in irregular glandular structures, some of which demonstrate intravascular permeation. (B) At higher magnification (200×), the tumor cells display marked pleomorphism, prominent nucleoli, and increased mitotic activity. (C–E) Immunohistochemical staining demonstrates strong and diffuse positivity for Cytokeratin 7 (CK7), Thyroid Transcription Factor 1 (TTF-1), and Napsin A, supporting the diagnosis of metastatic adenocarcinoma of pulmonary origin.

**Figure 3:**
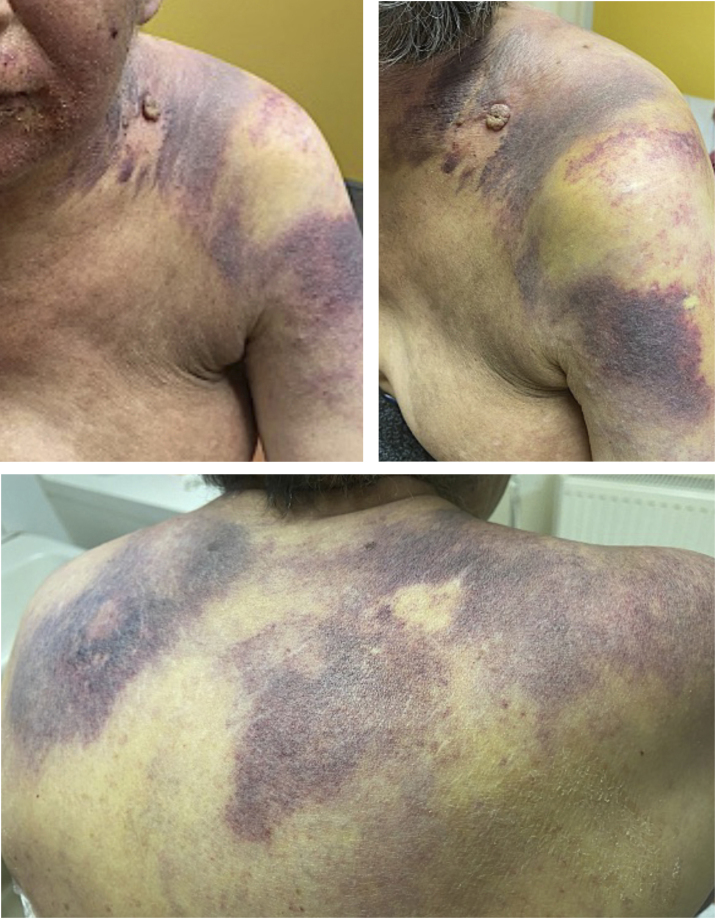
cutaneous improvement with diminution of the violaceous plaques in the upper body and face after starting Erlotinib.

Cutaneous metastases, although rare, indicate systemic dissemination and are usually associated with a poor prognosis, with a median survival of less than one year.^1^ CM is developed primarily by vascular invasion (lymphatic or hematogenous), caused by a complex phenomenon that occurs simultaneously with the development of the primary tumor.^5^ The most frequent tumor that metastasizes to the skin is melanoma, followed by breast tumors (24%), Kidney (4%), ovary (3.8%), bladder (3.6%), lung (3.4%), colorectal (3.4%), and prostate (0.7%) cancer.^6^ Regarding lung cancer, its main localizations of CM are the anterior thorax, abdomen, face and neck.^7^ Clinically, CM can exhibit a pleomorphic presentation, with dermal nodules being the most frequently observed manifestation.^1^, ^3^ They generally tend to appear near the primary tumor site, although this is not always the case.^1^, ^3^ They can mimic benign and malignant tumors, multiple dermatoses, and vascular lesions, debuting as inflammatory plaques, fibrotic, sclerodermiform, vasculitis-like lesions, keratoacanthomas, and telangiectatic granulomas, among others.^3^ A special and very unusual type of CM is hemorrhagic carcinoma. This is an inflammatory subtype of CM characterized by violaceous, purpuric, and indurated plaques that, upon histology, show infiltration of tumor cells in blood vessels, lymphatics, or both.^4^ To date, it has only been described in a limited number of patients with primary tumors in the breast and salivary duct.^4^ When possible, surgical resection is the treatment of choice, as it reduces tumor burden, improves functionality, quality of life, and even, partially, survival.^3^ In cases where surgery is not feasible, a combination of therapies applied directly to the skin and immunotherapy has proven to be more effective than each treatment alone.^3^ A meta-analysis by Spratt and colleagues reported high response rates and low recurrence rates following the use of skin-directed therapies.^8^ Among the available modalities, which include electrochemotherapy, photodynamic therapy, radiotherapy, intralesional therapy, and topical therapy, electrochemotherapy stands out as the most effective.^3^, ^8^ In our case, the use of BT alone yielded excellent results, reducing lesion extent, infiltration, and symptoms. Following the introduction of Erlotinib, a therapeutic synergy was achieved, optimizing the response. To our knowledge, this is the first reported case of cutaneous metastasis from pulmonary adenocarcinoma with hemorrhagic morphology. This case is important because it underscores the need for dermatologists to be aware of the various manifestations of cutaneous metastases, which could improve clinical management and therapeutic response.

## ORCID ID

Nelson Lobos-Guede: 0000-0003-0818-023X

Dan Hartmann: 0000-0002-7140-4294

Felipe Carvajal Villarroel: 0000-0002-2102-7750

Paloma Matus Concha: 0000-0002-6081-276X

Catalina Silva-Hirschberg: 0000-0002-4276-1284

Magdalena Delgado Barros: 0009-0008-1572-8439

## Research data availability

Does not apply.

## Financial support

This research did not receive any specific grant from funding agencies in the public, comercial, or not-for-profit sectors.

## Authors’ contributions

Nelson Lobos-Guede: Approval of the final version of the manuscript; critical literature review; intellectual participation in propaedeutic and/or therapeutic management of studied case; manuscript critical review; preparation and writing of the manuscript.

Dan Hartmann: Approval of the final version of the manuscript; critical literature review; manuscript critical review; preparation and writing of the manuscript.

Felipe Carvajal Villarroel: Intellectual participation in propaedeutic and/or therapeutic management of the studied case; manuscript critical review; preparation and writing of the manuscript.

Paloma Matus Concha: Intellectual participation in propaedeutic and/or therapeutic management of the studied case; manuscript critical review; preparation and writing of the manuscript.

Catalina Silva-Hirschberg: Intellectual participation in propaedeutic and/or therapeutic management of the studied case; manuscript critical review; preparation and writing of the manuscript.

Magdalena Delgado Barros: Intellectual participation in propaedeutic and/or therapeutic management of the studied case. Manuscript critical review; preparation and writing of the manuscript.

## Conflicts of interest

None declared.
